# Distribution of Chemical Species in the Water-Soil-Plant (*Carya illinoiensis*) System near a Mineralization Area in Chihuahua, Mexico—Health Risk Implications

**DOI:** 10.3390/ijerph15071393

**Published:** 2018-07-02

**Authors:** Angélica Cervantes-Trejo, Carmelo Pinedo-Álvarez, Eduardo Santellano-Estrada, Leonor Cortes-Palacios, Marusia Rentería-Villalobos

**Affiliations:** Departament of Natural Resources, Facultad de Zootecnia y Ecología, Universidad Autonoma de Chihuahua (UACH), Perif. Fco. R. Almada Km 1, 31453 Chihuahua, Mexico; angelica.cervantes@cimav.edu.mx (A.C.-T.); cpinedo@uach.mx (C.P.-Á.); esantellano@uach.mx (E.S.-E.); lcortes@uach.mx (L.C.-P.)

**Keywords:** toxic elements, transfer factor, bioaccumulation, hazard risk quotient

## Abstract

The aim of this study was to quantify major and trace elements in the water, soil, and plants (*Carya illionensis*) in an agricultural area; and to determine the health risks associated with the walnuts ingestion by calculating the risk quotient. Samples of water, soil, tree leaves, and walnuts were collected; in total, 135 samples were analyzed. Physicochemical parameters were obtained in irrigation water and soil samples. Elemental measurements were performed in an ICP, -OES and -MS. In addition, the distribution coefficient (soil–water), transfer factor (soil–plant), and hazard quotient were evaluated. In the irrigation water, As, Cr, and Pb, showed concentrations above the maximum allowable limits. Likewise, high concentrations of As, Cr, Pb, and Sb were found in tree leave samples, indicating a possible tendency of hyperaccumulation of those elements. Furthermore, Cr concentrations in walnuts were high by far than the reference value (FAO/WHO). A possible competition between chemical congeners were detected from transfer factors. Although, Sb concentrations in walnuts were also high, and no legislation for it in fruits exists. The hazard risk quotient for Sb did indicate a potential health risk. Finally, it is important to consider that the health risk increases when exposure through consumption takes place over a prolonged period of time, even in low concentrations.

## 1. Introduction

The occurrence and distribution of trace elements, even in low concentrations, represents a risk for ecosystems and human health [1]. The composition of these elements in nature depends on their local mineralization, interactions with the environment, soil, and water, and the specific biota of each site [2]. The environmental problems caused by trace elements are the result of their natural mobilization, lithology, weathering reactions, biological activity, volcanic emissions, rock solubilization, and anthropogenic activity [3,4]. Trace elements are directly emitted into the atmosphere and the soil, due to the emission of mining residues, industrial emissions, the use of pesticides and fertilizers, fossil fuels, and wood preservatives [4]. Trace metal contamination of food is one of the main aspects of food quality [5,6].

Heavy metals are the most dangerous because they are not chemically or biologically degradable, and because of the accumulation in living organisms [7,8]. The toxicity of trace elements varies from one element to the other. The accumulation of heavy metals has been reported to produce carcinogenic, mutagenic, and teratogenic effects—which are associated with cardiovascular, kidney, and osseous diseases—and nervous system disorders that may lead to harmful effects on the circulatory, enzymatic, endocrine, immunological, and central nervous systems [9,10].

The distribution of trace elements in nutrients that results from transformation, retention, and transport processes is different for every vegetable species. In the soil, the concentration of these elements is regulated by interrelated processes, including organic and inorganic complexation, oxidation-reduction reactions, precipitation–dissolution reactions, and absorption–desorption reactions [11]. These processes depend on the different chemical and environmental conditions, such as pH, electric conductivity, study site, geology of the agricultural soil, and degradation of the organic matter, among other processes [12]. The potential of plants to absorb trace elements and nutrients depends on the bioavailability of these elements in the soil, the age of the vegetal species, and the capacity of the plant to absorb, transport, and accumulate the elements in their roots and aerial parts, which is different for every vegetal species [5,9,13]. Therefore, it is necessary to identify the contaminants to evaluate their potential sources [14].

In Aldama, Chihuahua, Mexico (northern Mexico), a U mining operation began in the 1970s in the district of Pena Blanca. Here, the exploitation process and associated water–soil interactions resulted in the release of potentially toxic elements. Because the area of the mine is located near an agricultural area, mainly walnut orchards, the exposure to uranium and other toxic elements is of particular concern. Specifically, there exists no information on the distribution of trace elements and nutrients in walnut trees. In order to minimize adverse effects, it is important to continuously monitor the levels of trace elements in food [6]. The aim of this study was to quantify major and trace elements in the water, soil, and plants (*Carya illionensis*) in an agricultural area in Aldama, Chihuahua; and to determine the health risks associated with the ingestion of walnuts by calculating the risk quotient for each of the elements under analysis.

## 2. Materials and Methods

### 2.1. Study Area

**Figure 1:**
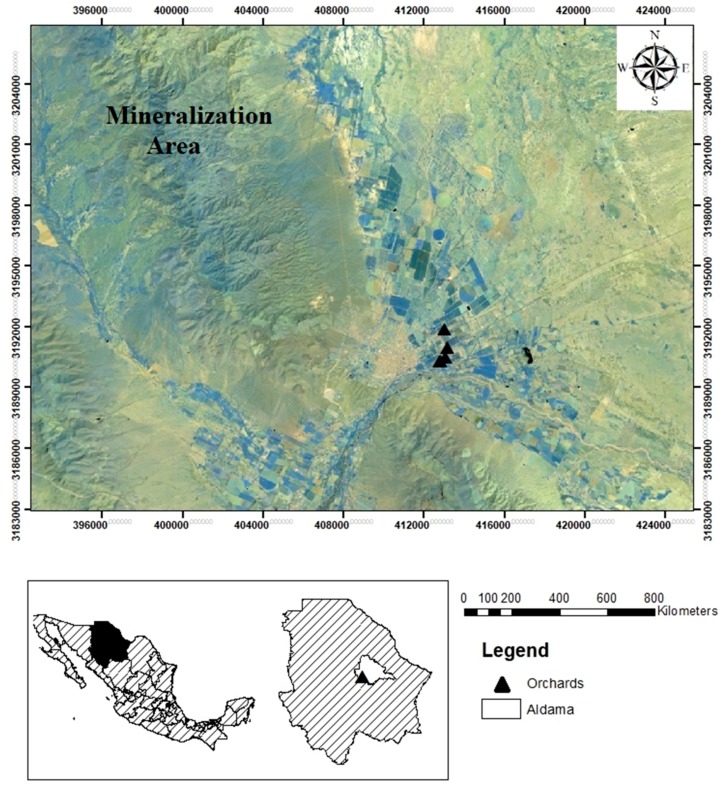
Location of the area of study and the sampling points selected in Aldama, Chihuahua.

The study area is located between latitude 28°50′00″ N and longitude 105°53′00″ W in the city of Aldama, Chihuahua, Mexico. In this area, semiarid conditions prevail with annual average precipitation of 336 mm. The Peña Blanca Uranium District is located to the west of the study area. The region is dominated by an alternating sequence of high country and watersheds that run from west to east. The Peña Blanca range developed from cratonic block towards the west during the Cenozoic Era. Specifically, it is located on a strip of hydrothermal uranium fields in a semiarid region from where a section of limestones, mudstones, sandstones, and tertiary rhyolitic tuffs originates that covers carbonated rocks that underwent tectonic deformations [15]; those rocks are found in a chemically oxidative and unsaturated zone, at 200 m over the water table. The local hydrology depends on the regional hydrological setting, and the groundwater system behaves as an unconfined aquifer [16]. In this region, the groundwater is the main water source for use and consumption by the population. The area of study and the sampling points are showed in Figure 1.

### 2.2. Sampling

The study took place in five walnut orchards: La Aurora, El Edén, Los Laureles, Santa Lucía, and Viña Grande. Sampling took place in October 2015 prior to harvest. Samples of water, soil, walnut tree leaves, and walnuts were collected in every walnut orchard; in total 135 samples were collected. Water was sampled from wells, except for Viña Grande where the irrigation is from surface water (river). Water samples were collected in a 1L container, where in situ parameters such as pH, electrical conductivity (EC), temperature (T), and total dissolved solids (TDS) were measured. Soil samples were taken from an area of 30 × 30 cm at a depth of 0–30 cm. Samples of 1 kg of soil, leaves, and walnuts were collected in a plastic bag for further analyses in the laboratory. In soil samples, pH, EC, and T parameters were measured. In addition, these samples were air-dried, crushed to a fine powder (200 mesh), and homogenized. Leaves and walnut samples were first thoroughly washed with tap water, and finally with deionized water, to remove impurities. In walnut samples, the shell was separated. Afterwards, samples of leaves, walnuts, and shells were ground in a glass mortar and sieved to obtain a fine powder. Finally, samples of soil, leaves, walnuts, and shells were dried in an oven to 75 ± 5 °C for 24 h.

### 2.3. Experimental Procedure

An aliquot of soil (500 mg), leaves (200 mg), shells (250 mg), walnut (250 mg), and groundwater (2 mL) samples was taken and transferred to a poly vial (tetrafluoroethylene, PTFE). Samples were subjected to acid digestion by using different reagents. For soils and leaves, 2 mL of HCl (36.5–38% J.T. Baker), 8 mL of HNO_3_ (65%), and 0.5 mL of H_2_O_2_ (30%, J.T. Baker) were added to the aliquots. For shells and walnuts, 4 mL of HNO_3_ (65.8%, J.T. Baker) and 2 mL of H_2_O_2_ were added to the aliquots. Finally, for groundwater 9 mL of HCl and 3 mL of HNO_3_ were added to the aliquots. Digestion of samples was performed using a microwave digestor (Multiwave GO, Anton Paar, Graz, Austria). Elemental measurements were performed in an ICP-OES (iCAP 6500, Thermo Scientific^®^, Waltham, MA, USA) and ICP-MS (NexION 300D, PerkinElmer^®^, Waltham, MA, USA). The standard solutions were prepared from 1000 mg/L of each element (National Institute of Standards and Technology, NIST; Gaithersburg, MD, USA) for detection by ICP-MS (iCAP 6500, Thermo Scientific^®^) and ICP-OES (NexION 300D, Perkin Elmer^®^). All measurements were performed in triplicates. The elements As, Ca, Cd, Cr, Cu, Fe, K, Mg, Mn, P, Pb, S, Sb, and Zn were obtained using ICP-OES, while U was measured by ICP-MS. The ICP-OES detection limit for elements analyzed is in the order of ppb (µg/L): As (1.4), Ca (0.58), Cd (0.05), Cr (0.15), Cu (0.69), Fe (0.25), K (0.6), Mg (0.3), Na (0.37), P (1.55), Pb (1.1), S (1.05), Sb (1.1), and Zn (0.19).

### 2.4. Distribution Coefficient (Kd)

The distribution and/or fractioning of chemical species between the solid-solution system is described by the distribution coefficient (kd) [17,18]. This coefficient expresses the amount of absorbed chemical species for every unit of mass of the solid, divided by the amount of the same chemical species dissolved in the liquid. In general, this is expressed by the equation [19]

kd = Cs/Cw,
(1)

where Cs is the element concentration in the soil (mg/kg) and Cw is the element concentration in water (mg/L).

Some factors that affect kd are; climatic conditions, soil characteristics, temperature, pH, ionic, and physical form of the chemical species, as well as the presence of other ions in the medium under study. The different ionic species are absorbed at different speeds; absorption of cations is generally stronger (large kd) compared to anions, due to the dominance of negatively charged particles on the surface of the solids.

### 2.5. Transfer Factors

The uptake of elements from soil to plants is measured by the transfer factor (TF). The TF for any given element can vary considerably depending on the kind of plant, as well as from one environment to another. The main parameters that modify the TF are; physical and chemical characteristics of the soil, trace element behavior in the soil and plant, and environmental changes [20,21]. The TF from soil to plants is calculated using the equation [22,23,24]

TF = C_plant_/C_soil_, (2)

where C_plant_ and C_soil_ refer to the concentration of each element in a specific vegetable or soil, respectively.

### 2.6. Statistical Analysis

In order to identify the statistical behavior of variables obtained, an analysis of variance, correlation analysis, and cluster analysis were performed. The procedure GLM (General Linear Model) in SAS 9 (Statistical Analysis System, 2002) (SAS institute, Cary, NC, USA) was used to obtain the estimators.

### 2.7. Hazard Quotients (HQ)

In order to estimate the risk from soil–water and vegetable ingestion, hazard quotients (HQs) were calculated using the following equations as reported by Warming [25].

HQ = EI/TDI,
(3)

where EI refers to the elemental intake of a particular trace element from a specific vegetable, soil, or water; TDI represents the tolerable daily intake values for each trace element (μg) per kg of bodyweight. EI was calculated using the equation

EI = (C × CR)/Bw,
(4)

where C refers to the concentration in either a specific vegetable, CR corresponds to the daily consumption of a specific vegetable, and Bw refers to the average bodyweight (72 kg).

## 3. Results

### 3.1. Concentration of Major and Toxic Elements in Soil and Water

Table 1 shows some physicochemical parameters measured in soil and water samples collected in the study area.

The water pH presented values with a tendency to neutral, with a maximum value of 8, whereas the soil showed pH values of 8.2 to 8.8. The EC in water showed higher values than in soil, from 0.2 to 1.1 mS/cm. In water, the TDS and hardness showed maximum values of 590 mg/L and 5249 mgCaCO_3_/L, respectively. The averaged heavy metal and trace metal concentrations in soils and irrigation water from five walnut orchards close the uranium district are shown in Table 2. It can be seen that the soil contains high concentrations of Fe, Ca, K, Mn, Na, P, S, and Zn. In addition, in water the elements with higher concentrations were Ca, Na, and S.

### 3.2. Concentration of Major and Toxic Elements in Carya illionensis

The averaged trace elemental concentrations in leaves, shells, and walnut samples from five walnut orchards close the uranium district are shown in Table 3. Results are the average concentration in every component of *Carya illionensis*.

From the results, it may observed that As an Pb were no found in walnut and shell. Likewise, Cu was not present in walnut. Cd was only detected in leaves and shells collected from La Aurora orchard. Elements with high concentration (>1000 mg/kg) in leaves were Ca > K > Mg > S = P, in walnuts were K > P > Mg > S > Ca, and in shell were only Ca.

## 4. Discussion

The study area contains soils with a slightly basic pH from 8.2 to 8.8 (Table 1), which can be attributed to the low annual precipitation in arid zones. Soil properties can influence the transfer of ions from the soil to the plant; previous studies indicate that the accumulation of heavy metals in the soil can be affected by the pH of the soil [28]. The hardness levels of the water do not present maximum allowable rates. However, the values that were found in the orchards under study are considered high; these values represent high salt concentrations in the water. The pH, EC and T values obtained from soil and irrigation water were statistically different.

The mobility of trace elements in the soil is complex and can be attributed to a variety of factors [3]: physicochemical properties, chemical species, climate, biological activity, and their interactions. The present study focused on the mobility of nutrients and trace elements with respect to physicochemical properties (pH, CE, analyte concentrations).

### 4.1. Element Characterization in Water

Trace elements such as As, Cr, Pb, and Sb are highly toxic to the environment. Moreover, the irrigation of soils with water that contains high concentrations of trace elements not only results in the transfer of these elements from the water to the soil, but also increases the bioavailability of these elements for plants [4]. In Table 2, it can be observed that the concentrations of these elements in the irrigation water that was analyzed in this study are above the recommended maximum concentration for irrigation water [26,27]. It was determined that the orchards La Aurora, El Eden, and Viña Grande presented As values of 4.2, 1.0, and 0.5 mg/L, respectively, which is higher than the maximum allowable concentration for irrigation water. It is known that in Argentina there is an important problem with the As concentrations in water. Some authors have reported values as high as 1.43 mg/L for water originating from a river [3]. Furthermore, in China As values of 0.01424 mg/L and 0.077 mg/L were reported in irrigation water from a river and a well, respectively [29]. These values are similar to the concentrations in this study. Moreover, the values in La Aurora are higher than these values. Cr was found in a range from 31.1 to 34.6 mg/L in the analyzed water samples. These values are higher than the maximum values allowed according to national and international legislations. The specie Cr (VI) is the most toxic specie, the concentration obtained in the present study is for total Cr. In irrigation water for agricultural areas, reported values of Cr range from 0.00236 to 0.31 mg/L [22,29]. Moreover, Cr values in irrigation water from a contaminated river in Argentina were found to vary between 1.67 and 11.51 mg/L [3]. The Cr values that were obtained in the present study are higher than the concentrations reported in those studies. Furthermore, in the orchard La Aurora the average Pb concentration was 11.5 mg/L, passing the established regulations for this element in irrigation water [26,27], whereas the Pb concentration in the orchard Los Laureles was 8.5 mg/L, passing the FAO regulation [27]. In related studies on the concentrations of Pb in irrigation water, maximum values of 0.00218 mg/L and 0.00435 mg/L were reported for river water and well water, respectively [29]. In the orchard La Aurora an average Sb concentration of 5.2 mg/L was found. However, there is no maximum allowable limit for Sb in the legislation for irrigation water. In other studies, Sb values of 0.00178 to 0.0052 mg/L have been reported for irrigation water [29,30,31].

Finally, another element for which the concentration was found to pass the established norm by FAO was Mn [32]. Mn does not present a risk to human health. However, high concentrations of Mn in irrigation water can be transferred to the soil, in this matrix they can oxidize and absorb arsenic species [33]. The Mn concentrations in irrigation water in the present study ranged from 0.3 to 0.9 mg/L; these values are higher than the maximum allowable concentration of 0.2 mg/L. Mn values in superficial irrigation water that have been reported in the literature are found in an interval of concentrations from 0.0446 to 0.219 mg/L [3,29,34], whereas in well water it was 0.458 mg/L [29]. Under arid conditions, chemical processes take place over a long time, due to low precipitation regime. However, groundwater is the main source for use and human consumption in the area, inducing to a high water extraction rate from aquifer. Consequently, this process might cause the increase of the toxic element contents in water. Thus, elements such as As, Cr, Pb, and Sb present in groundwater results in increased health risk for the inhabitants.

### 4.2. Element Characterization in the Soil

Currently, there is no legislation for nutrients and trace elements in the soil. However, the reference values for these elements can be obtained from their concentrations in the earth crust; these concentrations may vary depending on the lithology of each specific area. The concentrations of trace elements in the soil that were found in the present study can be considered high in comparison to published studies. The results in Table 2 show high concentrations of Ca in comparison to the average in the earth’s crust (4.1%), which is in agreement with the calcareous soil type of the region [35]. Calcareous soils are usually poor in organic matter content, N, Fe, P, and Zn [27].

In the study region, some elements in stream sediments were determined by the Mexican Geological Service (SGM) [36]. In Table 4, a comparison of the concentrations of As, Cu, Pb, Sb, and Zn is shown between what was published by SGM and what was found in the present study.

With the aim of determining if significant differences exist between the mean concentrations shown in Table 4, a Student’s *t*-test was performed (95% confidence level). The results show that there are no significant differences between the means of the analytes. Thus, the values found for these analytes in the present study correspond to what was reported for soil samples by SGM.

Furthermore, some authors obtained Cr concentrations between 26 and 708 mg/kg in agricultural soils [5,22]. The Cr in the soil in the present study falls within the range of these reported values, with a range of concentrations from 38.9 to 48.3 mg/kg. Concentrations of Sb in the soil were found in a range from 6.4 to 8 mg/kg, which is low compared to 486 mg/kg that was reported for a contaminated agricultural area [37]. In the present study, Mn in the soil was found to range from 375.2 to 509.8 mg/kg. A study in a potato agricultural area reported Mn values from 271 to 754 mg/kg [13].

The contents of Fe in the soil is higher than the average of 5% in the earth’s crust [35], which indicates an enrichment of this metal due to the use of crop fertilizers or additives. This is in agreement with results for soils in arid environments, where an average weight of 3% has been reported [38]. In addition, these authors report Ca concentrations of up to 11% weight in the soil, with a Fe/Ca ratio of 0.2, whereas in the present study this ratio is 2.5.

Furthermore, the only correlation trend that was observed in this matrix was between Ca and Pb (r = 0.75). Both elements have an oxidation state of 2+, for which it is suggested that these elements are present in the form of carbonates and sulphides in the soil.

### 4.3. Distribution Coefficient (Kd) of Trace Elements in the Soil/Water

The results for kd indicate that the majority of elements found here are mainly present in the soil (As, Ca, Fe, K, Mg, Mn, P, Pb, and Zn). These elements are mainly associated with oxides/hydroxides or in silicate matrices [39]. However, elements with a kd < 1 were mainly found dissolved in water; Cr, Na, and P, indicating the presence of these elements as ions originating from leaching from the soil. Finally, Sb and U were only detected in the soil.

### 4.4. Element Characterization in Carya illionensis

The determination of trace metals in the environment and food is an important task for scientists, environmentalists, and nutritionists. Similar to water and soil, foods may also be contaminated with trace metals, due to the increased use of chemical compounds in the industry, mining activities, and fertilizers, among others. The uptake of trace elements, including the natural radionuclides, by plants is of high importance in assessing the pathways of these elements into the environment. The transfer of trace elements may take place in the systems soil–plant and/or water–plant. For the human diet, trace metals can be classified as essential (Fe, Mn, Cu, Zn, and Se), probably essential (Ni, V, and Co) and potentially toxic (As, Cd, Pb, Hg, and U). Potentially toxic elements can be extremely harmful, even in low concentrations, when they are ingested for a prolonged period of time. Furthermore, essential metals may also create toxic effects when their intake is very high [40].

#### 4.4.1. Concentration of Major and Toxic Elements in Leaves

In general, the elements present in leaves were found in the following order of abundance (Table 3): Ca, K, Mg, S, and P > 1000 mg/kg; Mn, Zn, Fe, Na, and Cr > 100 mg/kg; Cu, As, and Pb > 10 mg/kg; and finally, Sb and U < 10 mg/kg.

The concentrations of As in the leaves were found in a range between 5.3 and 14.6 mg/kg; however, in the orchard La Aurora this analyte was not detected. In the soil, a high pH solubilizes As and it can be available to plants [41]. Hence, the As concentrations in leaves are higher than those in the soil in the present study. Several authors report a wide interval of As concentrations in tree leaves, from 0.54 to 190 mg/kg [3,42,43]. The values in walnut tree leaves reported in the present study are higher than the majority of As values in leaves of other vegetal species. In addition, Cr concentrations in walnut tree leaves in the present study are found in a range from 53.3 to 75.7 mg/kg. However, orchard La Aurora has a mean Cr concentration of 2.5 mg/kg. Cr values in tree leaves reported in the literature are found in a range of 4.7 to 13.8 mg/kg. Pb concentrations that have been reported for tree leaves go from 3.8 to 21.5 mg/kg [42,44]. The values of As, Cr, and Pb in walnut tree leaves are similar to those reported in the literature for different trees.

Another trace element that is found in a high concentration in the walnut tree leaves in the present study is Sb, ranging from 0.5 to 9.1 mg/kg. It is important to note that there is little information on Pb in the literature. One study in an agricultural area contaminated with antimony reported Sb concentrations of 0.31–1 mg/kg in corn leaves, 0.17–0.80 mg/kg in carrot leaves, and <0.02–0.07 mg/kg in leaves from a sugar plant [37]. Taking this into account, the Sb values that were found in walnut tree leaves in the present study can be considered high, passing the Sb values in contaminated sites.

Elements such as Ca, Fe, Mg, and Na are nutrients and micronutrients for the development of the walnut tree. Therefore, as the tree grows, there is an increased transfer of these elements to the aerial parts of the tree. In this regard, results for trace elements such as As, Cr, Pb, and Sb in walnut tree leaves show a correlation trend between the age of the trees and the presence of these elements in the leaves. This may also be attributed to the walnut trees being located in a semiarid region with high concentrations of salts in the soil, mainly CaCO_3_. High concentrations of carbonates limit the availability of these micronutrients, for which a common management practice is the addition of Fe, P, N, and Zn via foliar irrigation [45]. The orchard La Aurora contains the youngest trees, with an average age of 20 years, in comparison to the other orchards in this study. Therefore, the low concentrations of trace elements can be attributed to the age of the trees. In contrast, Cd and Cu concentrations were higher in younger trees.

#### 4.4.2. Concentration of Major and Toxic Elements in Shells and Walnuts

Nuts are both cholesterol-free and rich in important nutrients including proteins, and unsaturated fatty acids [46]. They also contain important micronutrients such as folic acid and niacin, vitamins (E and B6), and minerals such as Ca, Mg, Mn, Cu, Fe, Zn, Se, P, and K [47,48,49].

In general, elements such as Cr, Cu, Fe, K, Mg, P, S, U, and Zn were mainly found in the walnut. In contrast, Ca and Sb were concentrated in the shell. Furthermore, Pb was not detected in the shell or the nut. In the shell, the Ca content was lowest in orchard La Aurora, with an average concentration of 4948 mg/kg. In contrast, the Ca concentration in the nut was the highest in these trees. This confirms that the age of the trees plays and important role in the metabolic processes of the plant. The behavior of the macronutrients and P and K, and the micronutrient Zn is similar to that of Ca.

Some values for major and trace elements have been reported for walnut shells. These authors found that the concentrations in the shell in percent of mass decreases in the following order K > Ca > Mg > Al = Fe > Na [48]. Furthermore, trace elements were found in the following order of abundance; Cu, Zn, Cr, As, Cd, Pb, and Sb (mg/kg). The concentrations of major and trace elements reported by these authors are higher than those determined in the present study; in addition, the order of abundance was different. There are reports of concentrations such as Mn and U in potato peel [13]. These authors found concentrations of 1.8 to 4 mg/kg and 0.0626 to 0.5696 mg/kg for Mn and U, respectively. Compared to this, the contents of Mn in the present study is higher, whereas the concentration of U is lower.

Table 5 shows a comparison of the nutrient values and trace elements that were found in walnuts in the present study together with values for walnuts from different parts of the world. In studies reported in the literature, As in walnuts is found below detection level. In contrast, in the present study the concentration of Cd was not detected whereas in the same matrix values up to 0.16 mg/kg were reported [50].

Furthermore, the concentration of elements—such as Ca, Cr, Fe, and Zn—was higher in the samples analyzed here. The contents of K, Mg, and P are of the same order as those reported in other studies. Pb is one of the trace elements that was below the detection level in walnuts.

The Cu values in the present study are lower than those reported by Muller et al. [51] and Momen et al. [52]. Cu is an essential element for plant physiological processes. However, if Cu is absorbed in excess it can be considered a toxic element that inhibits growth [53]. In the present study, the Cu contents in walnuts presented values up to 6.3 mg/kg in orchards such as Los Laureles and Viña Grande, passing the maximum allowable concentration for Cu in fruits (Table 3).

Concentrations of trace elements have been reported for several fruits and vegetables. One example of this is the contents of As in potato and eggplant, with values of 0.01 and 0.2 mg/kg, respectively [54,55]. Furthermore, a study in Bangladesh reported a mean As concentration of 0.2 mg/kg in vegetables [54]. In both studies, the As contents in fruits and vegetables are lower than those analyzed here. In addition, Cu concentrations of 0.946 and 7.891 mg/kg have been reported for banana and mango, respectively [9]; which are similar to those determined in walnuts. Furthermore, Cu in walnuts analyzed here is nine times (8–10 mg/kg) higher than the maximum allowable concentration [56], in agreement with concentrations reported by Shaheen et al. [10] for bean (1.11 mg/kg) and carrot (0.296 mg/kg). Finally, a study in an area contaminated by a U mine [5] reports lower Cr values than here, found in tomato (1 mg/kg), banana (1.5 mg/kg), and papaya (2.4 mg/kg).

Concentrations of K found in walnuts in the present study range from 3925.6 to 5794 mg/kg. In the literature, the highest values reported for this element are in tomato (31,900 mg/kg), parsley (46,100 mg/kg), and cucumber (45,600 mg/kg) [22]. The S contents in walnuts in the present study was lower compared to concentrations of 1760 and 2698 mg/kg in hazelnut and almonds, respectively [56].

For vegetables such as corn, cabbage, spinach, and parsley, Sb concentrations of 0.58, 0.17, 0.043, and 0.24 mg/kg have been reported [37], which are lower than the concentration in walnuts in the present study. Additionally, U concentrations have been higher in foods such as wheat, 0.125 mg/kg; and onion, 0.113 mg/kg [20]; whereas in watermelon [57], pepper, eggplant, and pepper [20] they have been <0.1 mg/kg. The latter are within the range of U concentrations found in the present study.

Zn is considered one of the essential elements for growth and fruit production in walnut trees. This element is important for the biochemical and physiological processes in this plant. The Zn concentration in walnuts in the present study ranged from 44.9 to 74.6 mg/kg, which is higher compared to several other vegetables: bean (4.75), onion (3.449), potato (3.019), and tomato (2.0 mg/kg) [9]. In addition, in areas severely contaminated with arsenic in Bangladesh, Zn values up to 50 mg/kg are reported for spinach and coriander [58].

### 4.5. Transfer Factors (TF)

**Figure 2:**
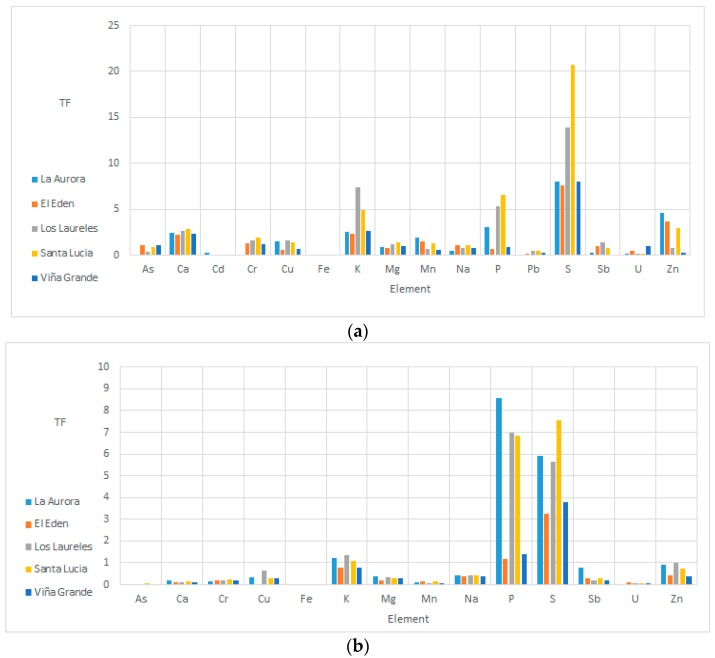
Transfer factors calculated: (**a**) from soil to leaves; and (**b**) from soil to walnut.

Transfer factors (TF) were calculated from soil to leaves and nuts. In Figure 2, transfer factors for soil–leaves and soil–walnuts are shown.The transfer factors (TF) for trace metals in walnuts decrease in the order; P > S > K > Zn > Sb > Na > Cu > Mg > Cr > Ca > Mn > U > As > Fe. P has the highest TF for all the walnut orchards, ranging from 1.19 to 8.58. This range is similar to a TF from 1 to 3.53 reported for tomato, parsley, and cucumber [22]. P does not present a human health risk because this analyte is an essential macronutrient for all living beings. Additionally, the TF for Zn from soil to walnut ranged from 0.3945 to 0.9901. These values are similar to TF values reported in the literate, from 0.2 to 5 in vegetables [4,22].

The TF of Cr in walnuts ranges from 0.1763 to 0.2543. This is higher than the TF reported for vegetables such as corn, eggplant, mint, pepper, tomato [4], and parsley [22], which does not exceed 0.03. The high TF for Cr from soil to walnut in the present study is important because of the high toxicity of Cr. Previous studies have established that Cr does not easily translocate within the plant, for which it is mainly concentrated in the roots. One of the most important factors is the pH, due to its effect on the solubility of the Cr species, and therefore its sorption in the soil and its bioavailability. When the pH is between 6 and 8.5, Cr is precipitated as Cr(OH)_3_ and is generally not available to the plant [4,11]. However, when the pH ranges between neutral and alkaline, the specie Cr^6+^ is present in very soluble forms, for example Na_2_CrO_4_ [59]. In the present study, the pH was between 8.3 and 8.8. Therefore, these alkaline pH values promote the solubility of Cr and its availability to the plant and the walnuts. The elements Ca, Fe, Mn, and U had a TF of ≪1, in other words, they do not have a high transfer from the soil to the walnuts.

The results for the TF of soil/leaves in walnuts indicates a high transfer of the nutrients Ca, Cu, K, Mg, Mn, P, S, and Zn. In the particular case of Cu, the TF ranges from 0.610 to 1.619. A study on spinach, cabbage, and celery leaves reported a mean TF of 0.02 [28]. The soil/leaves TF indicates a high transfer of As in the orchards El Edén and Viña Grande, with a TF of 1.097 and 1.137, respectively. This could be the result of the age of the trees, since these orchards have the oldest trees (approximately 60 years). In contrast, in the orchard La Aurora the TF for As is very low, because here the trees are younger. A study in vegetable leaves (spinach, celery, cabbage) reported TF values of 0.01 for As [28].

It is important to note that the behavior of Cd is opposite to that of the trace elements As, Cr, Pb, and Sb. In the orchard La Aurora, Cd has a TF of 0.262, in contrast to the low TF in the orchards with older trees. Possibly, Cd is inversely correlated with tree age. Therefore, it is suggested that Cd is only transferred to the leaves when the tree is younger and the plant has a higher need for nutrients for its growth.

Moreover, in the leaves positive correlations were found between Ca and Pb, Cr and P, and Cr and Fe (*P* < 0.05). This behavior could be explained on the basis of chemical congeners. Taking into account the oxidation states and atomic radii of the elements; the plant could be taking Pb (2+) as a chemical congener to Ca^2+^, since their atomic radii are very similar (~1.8 Å). Similarly, Cr and Fe have oxidation states of 3+, with atomic radii of 1.4 Å. Moreover, because the concentration of Cr in the leaves is higher than the concentration of Cr in the soil, it is suggested that the walnut tree leaves behave as hyperaccumulators of this analyte.

### 4.6. Hazard Risk Quotients (HQ)

In Table 6, the HQ are presented for the elements that were analyzed, as well as a comparison with values reported in several studies. This was determined for a consumption of 30 g per day. The HQ decreases in the following order; Sb > As > Mn > Fe > Cr > Zn > Cu > P > Mg > Ca > K > Na. When the HQ is equal to or higher than one, the analyte presents a health risk. The HQs of Sb, Cr, As, and Zn are representative indicate a health risk due to their high concentration in walnuts and their toxicity. Furthermore, these elements are associated with cancer risk [10]. A high HQ is observed for As, with a value of 0.1250. This value does not represent a health risk. However, it is important to note that the prolonged ingestion of As may pose a health risk.

The HQ for Sb was 1.23. In the literature there are no studies on HQs for Sb. The HQ that was determined for As in walnuts is slightly lower than the HQs reported by Noli and Tsamos [22] for tomato (<0.143), parsley (0.353), and cucumber (0.222). It is important to note that this study took place in an area close to an electric power plant that, because of the ash produced, has contaminated the environment. Furthermore, a study on fruits and vegetables reported HQ values of 0.032 for mango, 0.130 for bean, 0.043 for carrot, 0.01 for onion, 0.023 for potato, and 0.043 for tomato [9].

In the present study, the HQ for Cr in walnuts was 0.0025. This value does not represent a health risk. A study by Noli and Tsamos [22] reported a HQ for Cr of <0.001 for tomato, 0.009 for parsley, and 0.020 for cucumber. Another study on fruits and vegetables in Bangladesh found HQs for Cr of 0.0004, 0.002, and 0.006 for banana, bean, and tomato, respectively [9]. Finally, a study in Algeria found HQ values for Cr of 0.000064 for cucumber, 0.00044 for tomato, and 0.0023 for potato [10]. It is remarkable that the HQ values in the present study correspond to the highest values for Cr.

### 4.7. Correlation Analysis

**Figure 3:**
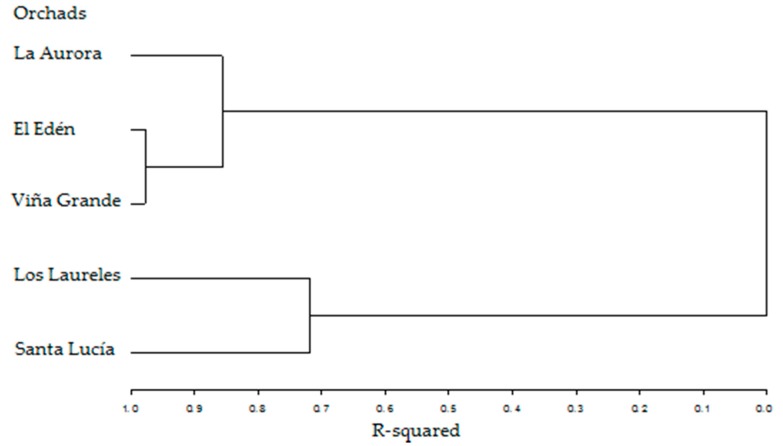
Tree diagram with the clusters of the orchards.

In Figure 3, the results of the Cluster Analysis that was performed with SAS 9.0 are shown, where the clustering of the orchards can be seen. It should be noted that this cluster analysis was performed with the data of the samples: nuts, shells, soils, leaves, and water of every orchard analyzed (El Edén, La Aurora, Viña Grande, Los Laureles, and Santa Lucía), with the analytes: As, Ca, Cd, Cr, Cu, Fe, K, Mg, Mn, Na, P, Pb, S, Sb, U, and Zn. Thus, 80 variables were analyzed.

In Figure 3, the first group that can be observed contains the orchards El Edén and Viña Grande, both orchards with trees of the same age (approximately 60 years old). A second cluster contains the previous orchards as well as La Aurora. This is attributed to the similarity in chemical soil characteristics of these three orchards. Finally, a third cluster combines the orchards Santa Lucía and Los Laureles, with trees that have an average age of 35 years.

## 5. Conclusions

In the present study, the distribution of trace elements and nutrients in the interaction water–soil–walnut tree was determined. In the irrigation water samples, As, Cr, and Pb, showed concentrations above the maximum allowable limits outlined in the Mexican legislation on irrigation water quality. Due to groundwater is used for human consumption, irrigation, and livestock feed a monitoring program and technologies to remove the contaminants are needed in order to improve the water quality.

High levels of As, Cr, Pb, and Sb were found in the walnut tree leave samples, indicating a possible tendency to hyperaccumulate these trace elements in walnut trees. These analytes solubilize from soil–plant, which is attributed to the high soil pH. Currently, the leaf litter is burned, however, it is important to implement adequate management practices for its final disposition.

The elemental transference obtained for the interactions water–soil, soil-leaves, soil–nut indicate possible chemical congeners when comparing to the chemical radio ionic properties and oxidation states, which is supported by the correlation analysis. This results in the uptake of Pb^2+^ instead of Ca^2+^, Cr^3+^ instead of P^3+^, and Cr^3+^ instead of Fe^3+^ by the plant.

The Cr concentrations in walnuts were up to 10 times higher than the reference value established by FAO/WHO. Sb concentrations in walnuts were also high. The hazard risk quotient was high for Sb, indicating a health risk. On the other hand, the hazard risk quotients for As and Cr did not indicate a potential health risk. However, it is important to consider that the health risk increases when exposure through consumption takes place over a prolonged period of time, even in low concentrations.

Finally, the trace elements could be transferred form the water to the soil and increase the availability of toxic elements to the walnut tree. Hence, it is suggested that the Mexican authorities establish a legislation with maximum allowable concentrations for trace elements in fruits and vegetables, to implement monitoring and evaluate health risks.

## Figures and Tables

**Table 1:** Chemical parameters measured in situ in the different soils and irrigation water of the orchards.

Orchard	Latitude	Longitude	pH		EC		TDS	Hardness
					(mS/cm)		(mg/L)	(mgCaCO_3_/L)
			Soil	Water	Soil	Water	Water	Water
La Aurora	28°51′12.29′′	105°53′30.93′′	8.66	7.62	0.15	0.88	450	3925
El Edén	28°50′20.94′′	105°53′38.18′′	8.18	7.55	0.15	1	500	4040
Los Laureles	28°50′27.57′′	105°53′29.06′′	8.79	7.7	0.16	0.18	590	5249
Santa Lucía	28°50′43.00′′	105°54′25.82′′	8.5	6.66	0.2	0.78	390	4771
Viña Grande	28°50′21.57′′	105°53′40.33′′	8.45	8.0	0.15	1.12	560	4161

**Table 2:** Averages of elemental content (µg/g) in soil and irrigation water, as well as their SD.

Orchard	La Aurora		El Edén		Laureles		Santa Lucía		Viña Grande		AL Irrigation	
Element	Soil	Water	Soil	Water	Soil	Water	Soil	Water	Soil	Water	ALa	ALb
As	7.7 ± 0.4	4.2 ± 0.2	13 ± 1.1	1 ± 0	12.7 ± 1.3	˂LOD	12.2 ± 0.4	˂LOD	12.8 ± 1.2	0.5 ± 0.01	0.4	0.1
Ca	6671 ± 491	1351 ± 112	8121 ± 238	1398 ± 79	6830 ± 389	1723 ± 131	6444 ± 311	1465 ± 172	8003 ± 265	1387 ± 117	-	-
Cd	2.7 ± 0.3	˂LOD	3.2 ± 0.3	˂LOD	2.4 ± 0.3	˂LOD	2.4 ± 0.5	˂LOD	3.1 ± 0.4	˂LOD	-	-
Cr	48 ± 1	31 ± 1	46 ± 3	34 ± 1	46 ± 4	34 ± 1	39 ± 2	32 ± 2	45 ± 1	35 ± 1	1	0.1
Cu	10.7 ± 1.1	˂LOD	21.2 ± 1.9	˂LOD	10 ± 1.4	˂LOD	7.5 ± 0.3	˂LOD	15.6 ± 1.7	˂LOD	-	-
Fe	17,753 ± 1372	181 ± 7	20,358 ± 787	188 ± 6	16,478 ± 1113	193 ± 11	17,897 ± 987	205 ± 22	19,482 ± 1642	181 ± 3	-	-
K	4682 ± 477	161 ± 21	5244 ± 480	34 ± 0.4	3332 ± 210	14 ± 5	3770 ± 160	28 ± 2	5109 ± 195	45 ± 5	-	-
Mg	3998 ± 469	135 ± 3	5751 ± 520	231 ± 2	4178 ± 159	272 ± 12	4487 ± 485	170 ± 12	5806 ± 209	159 ± 24	-	-
Mn	499.4 ± 9	0.9 ± 0.1	508.8 ± 33.8	0.4 ± 0	375.2 ± 11.2	0.3 ± 0.004	396.1 ± 32.3	0.3 ± 0.1	509.8 ± 15	0.9 ± 0.002	-	0.2
Na	492.4 ± 46	895 ± 12	520.8 ± 38	892 ± 9	505 ± 47	975 ± 59	500 ± 10	901 ± 102	516 ± 43	1507 ± 42	-	-
P	383.5 ± 11.7	7.5 ± 1.1	2256 ± 183.9	˂LOD	437 ± 33.5	5.6 ± 0.3	411 ± 89.5	8 ± 0.1	2178 ± 32.7	14 ± 1	-	-
Pb	˂LOD	11.5 ± 8.2	45.2 ± 5	˂LOD	28.1 ± 2.4	8.6 ± 0.2	30.6 ± 2.7	4.5 ± 0.3	47.4 ± 2.7	˂LOD	10	5
S	195 ± 1	375 ± 10	304 ± 18	361 ± 5	202 ± 14	442 ± 4	135 ± 29	291 ± 15	300 ± 14	371.5 ± 34	-	-
Sb	6.8 ± 1.4	5.2 ± 2.8	7.9 ± 1.6	˂LOD	6.4 ± 1.2	˂LOD	7 ± 1.5	˂LOD	8 ± 1.1	˂LOD	-	-
U	1.5 ± 0.1	˂LOD	0.407 ± 0.02	˂LOD	0.997 ± 0.06	˂LOD	0.861 ± 0.03	˂LOD	1.291 ± 0.09	˂LOD	-	-
Zn	82.4 ± 0.3	0.5 ± 0.01	146.7 ± 7.8	2.7 ± 0.3	61.5 ± 3.1	˂LOD	60.6 ± 8.1	2.7 ± 0.08	148.5 ± 5.5	0.9 ± 0.01	20	2

ALa = Allowed limit by CONAGUA [26]. ALb = Allowed limit by FAO [27]. <LOD = result lower than the limits of detection of ICP-OES/ICP-MS. - there is not allowed limit stablished by agencies.

**Table 3:** Averages of elemental content (range; mg/kg) in leaves, walnut, and shells samples.

Element	Leaves	Walnut	Shell
As	8.9 (0.001–14.6)	˂LOD	˂LOD
Ca	17,914 (16,301–18,721)	967 (769–1466)	6175 (4948–6796)
Cr	53 (2.5–76)	8.9 (8–10)	8.8 (1.5–16.2)
Cu	13 (10–16)	3.4 (0.5–6.3)	˂LOD
Fe	551 (282–669)	80 (75–90)	47 (24–73)
K	16,166 (11,997–24,541)	4457 (3926–5794)	2675 (1969–3586)
Mg	5010 (3691–6275)	1423 (1268–1579)	430 (337–574)
Mn	553 (241–936)	53 (33–73)	43 (19–70)
Na	433 (232–563)	206 (198–217)	203 (130–243)
P	1933 (1186–2685)	2981 (2696–3293)	270 (140–395)
Pb	10 (5–14)	˂LOD	˂LOD
S	2375 (1559–2806)	1090 (992–1155)	159 (129–186)
Sb	5 (0.5–9.1)	2.5 (1.3–5.3)	3.2 (0.001–5.8)
U	0.4 (0.1–1.3)	0.042 (0.001–0.1)	0.041 (0.001–0.1)
Zn	237 (44–536)	61 (45–75)	4.1 (1.1–6.9)

<LOD = result lower than the limits of detection of ICP-OES/ICP-MS.

**Table 4:** Comparison of soil ranges, present study and SGM values.

Element	Concentration of Present Study (mg/kg)	Concentration Found by SGM (mg/kg)
As	7.7–13	10.33–14.46
Cu	7.48–21.23	6.15–13.31
Pb	0–47.73	31.88–43.43
Sb	6.44–7.95	1.35–20.02
Zn	60.55–148.83	48.77–96.84

**Table 5:** Concentration of heavy metals and trace elements reported for walnuts in various parts of the world (literature values). Average concentration (µg/g) ± standard deviation.

Element	Spain [50]	Brazil [51]	Greece [52]	Present study	LP ^a^
As	<LOD	<LOD	-	1.05 ± 0.07	0.05
Ca	507 ± 63.5	-	-	842.97 ± 50.81	
Cd	0.16 ± 0.0198	<LOD	<LOD	<LOD	
Cr	1.801 ± 0.151	-	0.53 ± 0.003	8.94 ± 0.9	1.0
Cu	4.9 ± 0.1	12.4 ± 1.08	17 ± 1.2	4.15 ± 0.78	4.5
Fe	21.5 ± 1.3	0.70 ± 0.006	45 ± 3.1	80.44 ± 7.07	
K	4366 ± 195	-	-	4457 ± 773.8	
Mg	1276 ± 59	-	1330 ± 41	1422.8 ± 133.4	
Mn	81.2 ± 2.6	26.3 ± 2.34	26.5 ± 2.3	52.6 ± 17.23	
Na	-	-	-	206.1 ± 7.74	
P	2636 ± 76.1	-	-	2980.6 ± 229.4	
Pb	<LOD	<LOD	<LOD	<LOD	
S	-	-	-	1089.8 ± 78.15	
Sb	-	-	-	2.54 ± 1.59	
U	-	-	-	0.052 ± 0.01	
Zn	25.5 ± 0.5	26 ± 2.15	22.6 ± 3.0	60.7 ± 10.73	

^a^ = permissible limit (FAO/WHO, 2011). <LOD = result lower than the limits of detection of ICP-OES/ICP-MS. - = Not calculated.

**Table 6:** Hazard risk quotients for walnut (HQ) and those reported in the literature (HQ*).

Element	C	Intake	TDI	HQ	HQ*	
	(µg/g)	(µg/kg/día)	(µg/día)			
As	1.05	0.4375	3.5	0.1250	T < 0.142	(Noli & Tsamos, 2016)
					Py = 0.353	
					C = 0.222	
Ca	842.97	351.24	1,000,000	0.0004	-	
Cr	8.94	3.73	1500	0.0025	T < 0.001	(Noli & Tsamos, 2016)
					Py = 0.002	
					C = 0.001	
					C = 0.00006	(Cherfi et al., 2015)
					T = 0.00044	
					P = 0.0023	
Cu	4.15	1.73	900	0.0019	C = 0.006	(Cherfi et al., 2015)
					T = 0.010	
					P = 0.203	
Fe	80.44	33.52	8000	0.0042	-	
K	4457	1857.08	4,700,000	0.0004	-	
Mg	1422.8	592.83	420,000	0.0014	-	
Mn	52.6	21.92	2300	0.0095	-	
P	2980.6	1241.92	700,000	0.0018	-	
Na	206.1	85.88	1,500,000	0.0001	-	
Sb	2.54	1.06	0.86	1.2306	-	
Zn	60.7	25.29	11000	0.0023	T = 0.018	(Noli & Tsamos, 2016)
					Py = 0.009	
					C = 0.020	
					C = 0.0009	(Cherfi et al., 2015)
					T = 0.0058	
					P = 0.0279	

C = Cucumber, T = Tomato, P = Potato, Py = Parsley, W = Walnut. - = not calculated.
